
JOURNAL INFORMATION
==============================
NLM Title Abbreviation: ChemistryOpen
Journal ID: ChemistryOpen
NLM Journal ID: 101594811
Journal ID: open

ChemistryOpen

EISSN: 2191-1363
Publisher: Wiley

ARTICLE INFORMATION
==============================
PMCID: PMC3922443
PMID: 24551504
DOI: 10.1002/open.201290017
Article version: 1
Subjects: Graphical Abstract

Graphical Abstract: ChemistryOpen 4/2012

Print publication date: 2012 Aug
Electronic publication date: 2012 Aug 28
Volume: 1
Issue: 4
First page: 155
Last page: 161
Copyright: Copyright © 2012 WILEY-VCH Verlag GmbH & Co. KGaA, Weinheim
Copyright year: 2012
License: This is an open access article under the terms of the Creative Commons Attribution Non-Commercial License, which permits use, distribution and reproduction in any medium, provided the original work is properly cited and is not used for commercial purposes.
License URL: https://creativecommons.org/licenses/by/2.5/

==============================
DOI: 10.1002/open.201200017

### Spatiotemporal Pattern Formation during Electrochemical Oxidation of Hydrogen on Platinum

Hamilton Varela*[a]

**Awarding Institution:** Freie Universität Berlin (Germany)
**Date Awarded:** November 12, 2003
**Supervisors:** Prof. Dr. Katharina Krischer, Physical Chemistry Department, Fritz-Haber-Institut der Max-Planck-Gesellschaft, Berlin (Germany)
Prof. Dr. Helmut Baumgärtel, Institut für Chemie, Freie Universität Berlin (Germany)

The study of spontaneous emergence of spatiotemporal patterns far from equilibrium or dissipative structures has become an increasingly growing interdisciplinary field of research. From a chemical point of view, the spatially varying properties are usually thought of as being composed of several individually reacting elements that are coupled among each other. Examples of well-studied systems are the celebrated Belousov–Zhabotinsky reaction, heterogeneous catalytic reactions, and electrochemical reactions. In all cases, pattern-forming systems are characterized by internal feedback loops and are mathematically described by nonlinear evolution equations.

Feedback loops form especially easily in electrochemical systems, and, in fact, any electrochemical system can in principle display temporal symmetry breaking in the form of spontaneous current or potential oscillations in some parameter range when driven far from equilibrium. Pattern formation in electrochemical systems occurs at the electrode–electrolyte interface and results from the interplay between interfacial kinetics and transport processes parallel to the electrode surface. The interfacial region exchanges energy and mass with the bulk electrolyte and the system is kept far from equilibrium by a constant supply of electrical energy from the surroundings to the electrode as schematically shown in Figure 1. This supply usually comes from a potentiostat or a galvanostat. In the galvanostatic operation mode, the potential of the working electrode varies in order to keep a preset current value. In the potentiostatic operation mode, the potential of the working electrode is controlled with respect to a reference electrode. In the scheme depicted in Figure 1, the electroactive species A diffuses from the bulk solution to the elec-

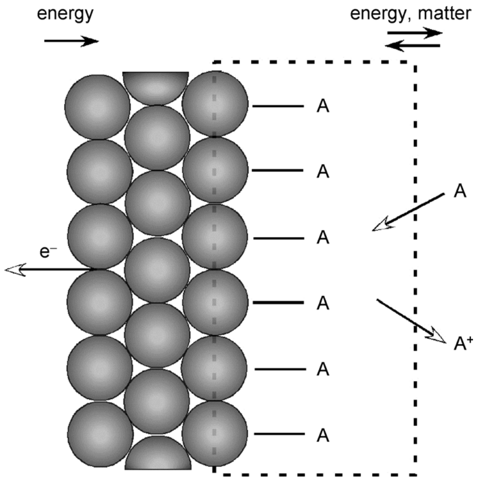

**Figure 1.** Schematics of the electrochemical interface as an open system in which electrons flow through the electrode–electrolyte interface and electroactive species flow through the reaction plane–bulk solution interface. The electro-oxidation of A ($A \rightarrow A^{+} + e^{-}$) is illustrated.

[a] Prof. Dr. H. Varela
Institute of Chemistry of São Carlos, University of São Paulo
CP 780, CEP 13560-970, São Carlos, SP (Brazil)
and
Ertl Center for Electrochemistry and Catalysis, GIST
Cheomdan-gwagiro 261Buk-gu, Gwangju 500-712 (South Korea)
E-mail: varela@iqsc.usp.br

The full thesis is available on the WWW; for access, visit http://dx.doi.org/10.1002/open.201200017.

© 2012 The Authors. Published by Wiley-VCH Verlag GmbH & Co. KGaA. This is an open access article under the terms of the Creative Commons Attribution Non-Commercial License, which permits use, distribution and reproduction in any medium, provided the original work is properly cited and is not used for commercial purposes.

trode, adsorbs at the working electrode, where it is then oxidized to $A^+$. Finally, the product $A^+$ desorbs and diffuses from the reaction zone to the bulk solution.

In spatially extended electrochemical systems, the individual elements composing the electrode are predominantly coupled through the electric potential in the electrolyte, and, in contrast to diffusion, the spatial coupling in electrochemical systems is long-range or nonlocal. The range of the migration coupling can be experimentally changed by varying the distance between the counter electrode (CE) and the working electrode (WE). The possibility of changing the range of the migration coupling from a long-range to a localized, diffusion-like coupling is a very interesting peculiarity that differentiates electrochemical systems from reaction-diffusion systems. In addition to migration coupling, a global coupling might be induced in electrochemical systems in both potentiostatic and galvanostatic operation modes. The global constraint is positive or synchronizing when the system is under glavanostatic control, and it is negative or desynchronizing under potentiostatic control, if a part of the cell resistance between the counter and working electrodes is partially compensated.

In this thesis several aspects of self-organization phenomena in electrochemical systems are experimentally studied. The first aspect concerns temporal self-organization phenomena and deals with the question how complex, or rather more precisely, higher periodic or aperiodic current responses might be in cyclic voltammetric experiments.

Other aspects deal with spatial pattern formation and cover two main problems, namely: a) pattern formation in the oscillatory region of the electrode reaction, whereby the spatial coupling is exclusively due to the electric field of the electrolyte (migration coupling), and b) the impact of an additional negative global coupling (NGC) caused by the external control of the electrochemical reaction by means of a potentiostat on pattern formation of oscillatory electrochemical systems.

The experiments on the complex voltammetric responses were carried out with hydrogen oxidation on a platinum electrode in diluted sulfuric acid as the electrolyte (i.e., the $Pt|H_2SO_4|H_2$ system). The experiments on the spatial pattern formation were performed using hydrogen oxidation reactions in the presence of chloride and copper ions (i.e., the $Pt|H_2SO_4,Cl^-,Cu^{2+}|H_2$ system).

In Chapter 4 the occurrence of complex voltammetric responses in the bistable $Pt|H_2SO_4|H_2$ system is presented. The observed periodic and aperiodic cyclic voltammograms are very similar to the ones previously reported during the electro-oxidation of small organic molecules on platinum and palladium under comparable conditions. It was shown that, in contrary to earlier beliefs, the mechanism underlying the complex response does not necessarily require the occurrence of any reaction between carbon-containing intermediates and surface oxide. Instead, the complex behavior results from the interplay between the negative differential resistance and the roughening process accompanying the surface oxide reduction followed by the relaxation of the rough surface. Theoretical simulations using a mathematical model comprising four ordinary differential equations further supported this conjecture. Considering the general nature of the conditions leading to the complex voltammetric responses, it was concluded that the observed phenomena should exist for a large variety of electrode reactions and electrode materials.

Chapter 5 launches the series of subsequent chapters on the spatiotemporal pattern formation in the oscillatory $Pt|H_2SO_4,Cl^-,Cu^{2+}|H_2$ system. In this chapter the system was studied in the absence of any global interaction. Hence, migration coupling, which is always present in electrochemical systems, was the only mechanism leading to a lateral communication along the ring electrode. The experiments were done for two distances between the CE and the WE, corresponding to two different ranges of the migration coupling. In both configurations, a transition from periodic spatiotemporal structures to highly disordered spatiotemporal states was observed when increasing the applied voltage. These findings present one of the very rare (if not the first) experimental evidences of a transition into chemical turbulence in oscillatory media. Moreover, the impact of the localization of the migration coupling on the patterns could be clearly proven. The more localized the coupling was, the more irregular were the spatiotemporal patterns, and the smaller were their characteristic wavelengths.

The studies on the impact of a global coupling on the dynamic behavior of the $Pt|H_2SO_4,Cl^-,Cu^{2+}|H_2$ system started with the experimental verification of the global coupling in presence of ohmic drop compensation. According to this theory, the NGC strength is only a function of the compensated electrolyte resistance and the total cell resistance. It does not depend on the manner in which compensation is achieved, whether a Haber–Luggin capillary or an electronic compensation is used. A comparison of the spatiotemporal dynamics obtained using a Haber–Luggin capillary to those obtained using a negative impedance device inserted between the WE and the potentiostat, led to an experimental confirmation of this prediction (see Chapter 6).

© 2012 The Authors. Published by Wiley-VCH Verlag GmbH & Co. KGaA, Weinheim

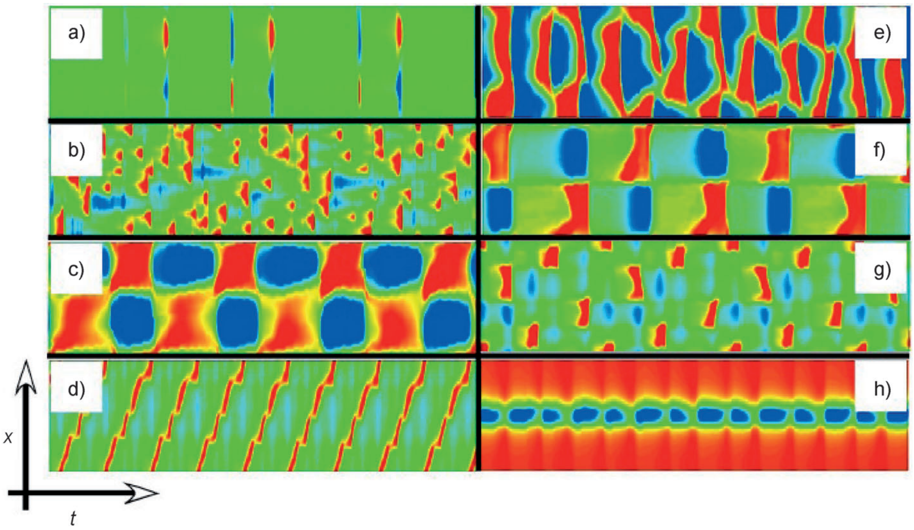

**Figure 2.** Examples of experimental spatiotemporal patterns obtained for the oscillatory electro-oxidation of hydrogen on a rotating platinum ring electrode. a) periodic modulations, b) electrochemical turbulence, c) two-phase clusters, d) travelling pulses, e) transition state between periodic and chaotic regimes, f) secondary-type of two-phase clusters, g) five-phase clusters, and h) trapped oscillations between potential walls. The spatial coordinate *x* represents the interfacial potential distribution along the ring electrode, that is, from 0° to 360°. The color scale is normalized in each plate and varies from blue to red with amplitudes between 200 mV (e.g., in panel a) and 1000 mV (e.g., in panel h).

The influence of the NGC strength on spatiotemporal pattern formation in the $Pt|H_2SO_4,Cl^-,Cu^{2+}|H_2$ system was studied in Chapter 7, which is divided into two parts according to the copper concentration used. At lower copper concentration, at which a turbulent behavior was observed in the absence of NGC, the turbulence could be suppressed even when only a weak NGC was applied. An increase in NGC strength led to the unusual observation: 2-PC of type I→2-PC of type II→IC→5-PC→ pulses (PC = phase clusters, IC = irregular clusters). To the best of our knowledge, we are not aware of other experimental observations of periodic five phase clusters (resulting from a global constraint). At higher copper concentrations, the spatiotemporal patterns include target patterns (as well as their asymmetric version) at intermediate coupling strength as well as modulated oscillations at low coupling strength and modulated pulses at high coupling strength.

Chapter 8 discusses the observation of composite spatiotemporal patterns arising from the interaction between two negative differential resistances (NDRs). Namely, in the presence of strong NGC and high applied voltages, one owes to the $Cl^-$ adsorption at less positive potentials and one owes to the oxide formation at more positive potentials. The emergence of such patterns takes place at potentials at which oxide formation begins. At these potentials and in the presence of NGC, the electrode can split into two different regions, one being covered by oxide, the other consisting of bare platinum. The novelty of the observed pattern is related to the development of spatiotemporal oscillations inside the active domain of the prepatterned stationary state due to the (HN-NDR) oscillatory $Pt|H_2SO_4,Cl^-,Cu^{2+}|H_2$ system. The observed scenario is expected whenever there is a succession of two NDRs in the current ($I$) versus double layer potential ($\phi_{DL}$) curve, as it is the case in many HN-NDR systems. Furthermore, the discussed mechanism should be a universal route leading to a substructuring of space in systems that experience a global coupling and possess at least two adjacent regions in parameter space that exhibit distinct dynamical instabilities, that is, two distinct positive feedback loops.

Figure 2 illustrates eight different examples of one-dimensional spatiotemporal self-organization during the oscillatory electro-oxidation of hydrogen on a rotating platinum ring under potentiostatic regime and different coupling conditions.

The results are also of general nature and present an experimental contribution towards the understanding of spatiotemporal self-organization phenomena. From a more general point of view, this work provides further insight into the understanding of the collective dynamics of a population of individual chemically active units that are coupled either exclusively by migration or by migration and a global feedback.

© 2012 The Authors. Published by Wiley-VCH Verlag GmbH & Co. KGaA, Weinheim

**Keywords:** complex systems · electrochemical systems · oscillations · spatiotemporal patterns · spatial coupling

**Publications arising from this work:**

- F. Plenge, H. Varela, K. Krischer, *Phys. Rev. E* **2005**, *72*, 066211.
- H. Varela, C. Beta, A. Bonnefont, K. Krischer, *Phys. Chem. Chem. Phys.* **2005**, *7*, 2429–2439.
- F. Plenge, H. Varela, K. Krischer, *Phys. Rev. Lett.* **2005**, *94*, 198301.
- H. Varela, C. Beta, A. Bonnefont, K. Krischer, *Phys. Rev. Lett.* **2005**, *94*, 174104.
- H. Varela, A. Bonnefont, K. Krischer, *ChemPhysChem* **2003**, *4*, 1348–1351.
- K. Krischer, H. Varela, A. Birzu, F. Plenge, A. Bonnefont, *Electrochim. Acta* **2003**, *49*, 103–115.
- H. Varela, K. Krischer, *J. Phys. Chem. B* **2002**, *106*, 12258–12266.
- P. Grauel, H. Varela, K. Krischer, *Faraday Discuss.* **2002**, *120*, 165–178.
- H. Varela, K. Krischer, *Catal. Today* **2001**, *70*, 411–425.

Published online on July 4, 2012

© 2012 The Authors. Published by Wiley-VCH Verlag GmbH & Co. KGaA, Weinheim
